# Neurologic Complication Due to Crystallization After Drug Interaction Between Alkalized Lidocaine and Ropivacaine: A Case Report and *in vitro* Study

**DOI:** 10.3389/fmed.2022.919911

**Published:** 2022-05-26

**Authors:** Afang Zhu, Lijian Pei, Wei Liu, Wencong Cheng, Yu Zhang, Yuguang Huang

**Affiliations:** Department of Anesthesiology, Peking Union Medical College Hospital, Chinese Academy of Medical Sciences and Peking Union Medical College, Beijing, China

**Keywords:** emergency cesarean section, epidural labor analgesia, delayed neurologic recovery, alkalized lidocaine, ropivacaine crystallization

## Abstract

**Background:**

For pregnant women transferred to emergency cesarean section after receiving epidural labor analgesia, there is still a debate over the effective and safe means of rapidly delivering surgical anesthesia. Alkalized lidocaine is often adopted for fast onset time; however, crystallization of the anesthetic may cause severe neurologic symptoms.

**Case Presentation:**

We report a case of a pregnant woman who underwent emergency cesarean section with satisfied analgesia but experienced severe weakness and paranaesthesia in the lower limb. After excluding lumbar disc herniation, obstetric nerve injury, and anesthesia technique causes by symptoms signs and magnetic resonance imaging, drug-related injury became the most likely cause. Our *in vitro* testing confirmed the obvious precipitation of additional anesthetic-concentrated ropivacaine (0.5–1%) with pretreated alkalized lidocaine. With trophic neurotherapy, the parturient attained prompt relief of weakness by day four, but delayed recovery of numbness, which lasted for 4 weeks.

**Conclusion:**

To date, this is the first case reporting neurologic complication possibly due to drug crystallization in cesarean section. Our study confirmed the rapid onset of alkalized lidocaine and its safety to pretreated routine labor dose of ropivacaine (0.09%). However, additional anesthetic-concentrated ropivacaine (0.5–1%) to maintain the anesthesia and analgesia level is not suggested.

## Introduction

Standard monitoring protocols for effective and safe anesthesia are important for parturients transferred for emergency cesarean section upon receiving epidural labor analgesia, even critical to the implementation of the three-child policy in China. Sodium bicarbonate was added to lidocaine mainly to adjust the pH for faster onset of action and enhance the depth of block (1, 2), which makes it an available choice for rapid anesthesia. However, the most commonly used drug in labor analgesia, ropivacaine can easily crystalize with pH elevation (3). We report a case of a pregnant woman who experienced severe weakness and paranaesthesia post-operatively, potentially due to ropivacaine crystallization. Differential diagnosis and various mixtures of alkalized lidocaine and ropivacaine are discussed.

## Case Description

A 33-year-old female, 168 cm height/100 kg weight, G1P0, at 40 weeks 6 days gestational age was admitted in latent labor with an uncomplicated pregnancy. She underwent labor epidural placement at L2–3 level smoothly, and attained satisfactory analgesia with 0.09% ropivacaine and sufentanil (5 mcg/mL) at 6 mL/h for labor. For intrauterine fetal distress, decision to proceed with emergency cesarean section was undertaken 6 h after labor analgesia. With a test dose of 3 mL 2% lidocaine after 3 min, 12 mL fresh 0.83% alkalized lidocaine (L_alk_) (15 mL 2% lidocaine plus 3 mL 5% sodium bicarbonate) was administered, followed by 7.5 mL 0.75% ropivacaine 10 min later to raise the anesthesia level from T9 to T6. Another 7.5 mL 0.75% ropivacaine was added 40 min later when closing the peritoneal. The parturient underwent cesarean section comfortably, and the epidural catheter was removed at the end of surgery. An infant with 3,990 g was delivered with right occipital transeverse position, and her Apgar score was 10 at 1, 5, and 10 min.

On the second postpartum day, the patient noted numbness and weakness in the knee when bearing weight on the right lower limb and fell when she stood, with no back or leg pain. Neurological pinprick examination identified decreased sensation in the lateral-middle lower-third of the thigh, lateral crus, and dorsum pedis, with muscle strength grade-2 and a weak knee reflex, no pathological signs elicited and negative straight leg elevation test. Magnetic resonance imaging (MRI) revealed minor expansion of the L2/L3 disc but no other abnormalities. The weakness and paresthesia improved by day three and four, respectively. She was discharged home on day five with mecobalamin and vitamin B1 for trophic neurotherapy, and the numbness persisted for 4 weeks.

Generally, the parturient received smooth labor analgesia and cesarean section, but presented with obvious weakness of the right lower limb. Although relief was observed by day four, she experienced slow recovery of numbness lasting 4 weeks, with negative magnetic resonance imaging or pathological signs.

## Discussion

Lumbar disc herniation often presents with low-back pain and lower limb pain; a positive straight leg elevation test and imaging performance is consistent with the symptoms. For this parturient, symptoms including weakness and numbness of the thigh seem to support herniation. However, the MRI showed no evidence of compression at the nerve root, namely herniation could not be responsible for her symptoms.

Lower extremity mononeuropathies and radiculopathies occur during delivery with an incidence as high as 0.92%, with the following nerves mostly affected: lateral femoral cutaneous nerve of the thigh, femoral, common peroneal, lumbosacral plexus, sciatic, obturator and lumbar or sacral root (4). Pregnancy-induced hormone, weight increase, articular ligament changes, and posture contribute to these. For posture, most parturients experienced trial of vaginal delivery and compression from fetal head especially during the second stage of labor. For this parturient, emergency cesarean section decision was made at three fingers-open, and she did not experience semi-Fowler lithotomy position, active force, and trial of vaginal delivery. In regard to childbirth factors, for one hand, the symptoms caused by fetal posture-related lumbosacral plexus or femoral injury are often foot drop, unable to stretch the leg or reduced knee flex, and the recovery of muscle force often takes few weeks or months. For another hand, the parturient's labor stagnated in the first stage, and her sensory and motor functions were normal before cesarean section.

**Figure 1:**
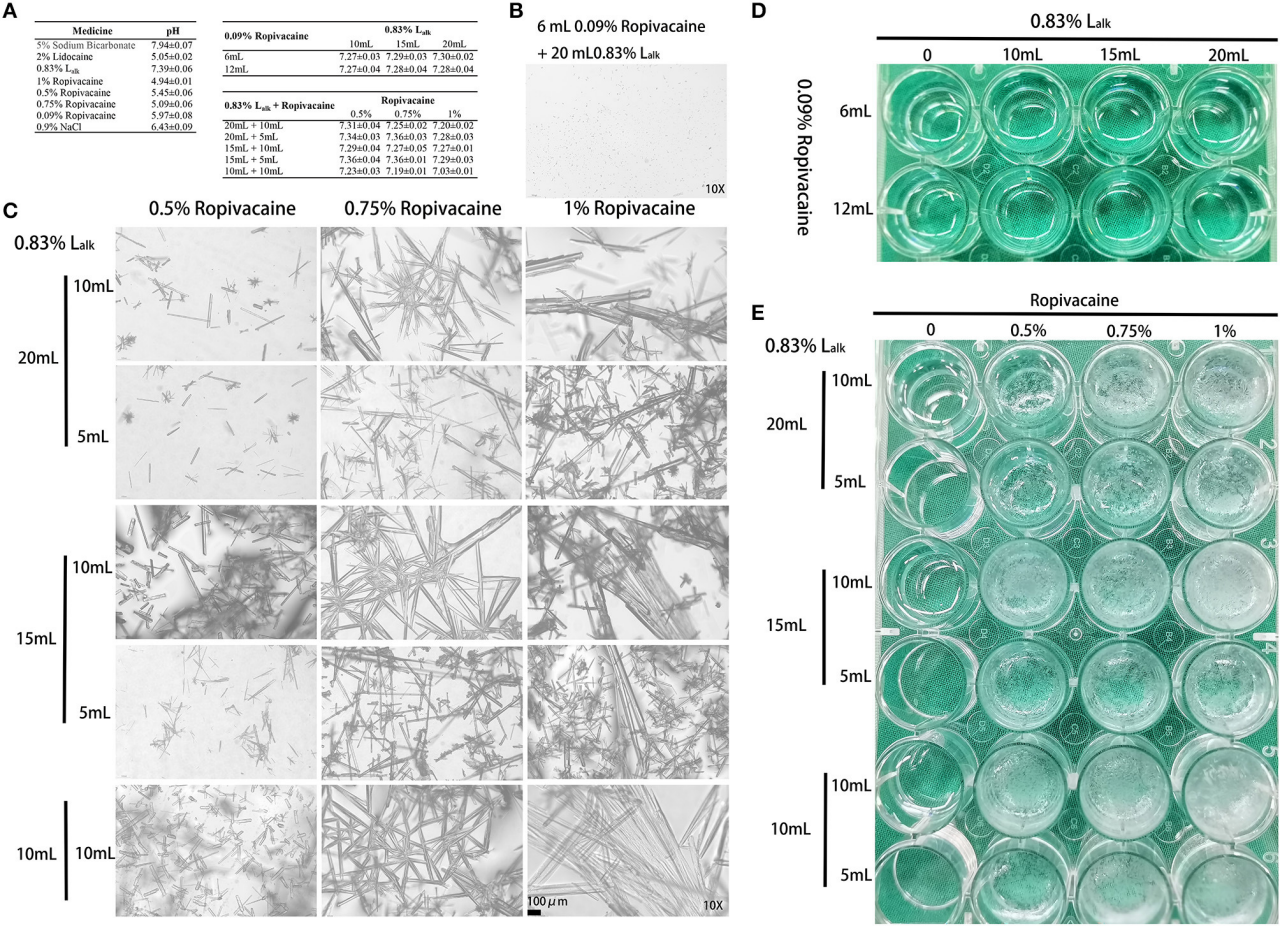
Crystallization of medicine. **(A)** The pH values of each medicine (mean ± SEM) detected by Sartorius PB-10 within 10 min (*n* = 3–6). **(B)** Microscopic image (10X) of the 6 mL 0.09% ropivacaine + 20 mL 0.83% L_alk_ group after mixing over 1 h. **(C)** Microscopic image (10X) of the 0.83% L_alk_ – ropivacaine (0.5–1.0%) mixture. **(D)** Macroscopic appearance of the 0.09% ropivacaine (routine labor analgesia) – 0.83% L_alk_ (routine doses). **(E)** Macroscopic appearance of the 0.83% L_alk_ – ropivacaine (0.5–1.0%) mixture. L_alk_, alkalized lidocaine.

Hence, factors from the parturient (herniation) and delivery (obstetric nerve injury) were not considered, and the MRI examination excluded epidural haematoma related compression. Could this abnormity be anesthetic-related? To test this hypothesis, *in vitro* testing was performed to assess the stability of the L_alk_ and ropivacaine mixture. We mixed sodium bicarbonate with lidocaine to adjust the pH for faster onset of action (~10 min). Despite its widespread use, ropivacaine has been reported to rapidly crystallize with pH elevation (3, 5–7). To verify crystallization, we mixed various doses of 0.83% L_alk_ with 0.09% or 0.5–1% ropivacaine at 37°C. Routine ropivacaine for labor analgesia is 0.09% with 6 mL as bolus dose and 6 mL/h as maintenance dose; thus, the selected doses of ropivacaine were 6 and 12 mL. A dose of 0.83% L_alk_ is recommended for cesarean section from 10 to 20 mL; and ropivacaine for cesarean section is 0.5% with 5 or 10 mL. Therefore, we designed two tests with different concentrations of ropivacaine and various doses to detect the mixed effects. All the medicine and materials were kept in a 37°C incubator 30 min prior to use, and each mixture was prepared freshly. The pH of each medicine and mixture were recorded within 10 min, and observed with 1 h. For the short-term stability of pH-buffering (8), this test was repeated six times in all. The pH values of each medicine and mixture are presented in Figure 1A, and the values of L_alk_ with ropivacaine ranged from 7.03 to 7.36. Both macroscopic and microscopic visualization observed within 1 h detected no crystals in the L_alk_ −0.09% ropivacaine mixture (Figure 1D). However, very slight crystallization could be observed under the microscope after mixing over 1 h (Figure 1B). Upon increasing ropivacaine from 0.5 to 1% (routine concentration for cesarean section), larger volumes and number of rod-shaped crystals were detected; when increasing the volume of ropivacaine from 5 to 10 mL, while decreasing the volume of L_alk_ from 20 to 10 mL, the rod-shaped crystals became much larger (Figure 1C). The macroscopic visualization revealed more precipitation with ropivacaine increasing from 0.5 to 1%, presented as a cloudy appearance (Figure 1E). With higher volume of ropivacaine and smaller volume of L_alk_, the occurrence of crystallization was easy with increase precipitation (Figure 1E). Collectively, we observed a ropivacaine concentration- not pH-dependent relationship precipitation. Milner et al. showed that ropivacaine (0.75 and 1%) was unsuitable for alkalization signals at pH of 6.0 (3). Actually, even at pH value of 5.8, crystals were visible under the microscope immediately in Colsoul et al.'s study who evaluated the physical stability of the mixture of ropivacaine, clonidine, and adrenaline tartrate (5). Adding corticosteroid to local anesthetics could also increase the pH of the mixture and may cause crystallization, which has been verified in Watkins et al.'s (6) and Hwang et al.'s (7) studies. Watkins et al. observed ropivacaine crystal formation when mixed with dexamethasone, and showed a positive correlation of crystallization with higher pH (7.0 vs. 6.8) and with higher concentrations of dexamethasone (10 vs. 4 mg/mL). In Hwang et al.'s study, when ropivacaine was mixed with dexamethasone or betamethasone, crystals larger than an arteriole at physiologic pH levels (7.0 and 7.5, respectively) were observed. All these studies suggested that we should pay attention to alkalized ropivacaine for its potential hazards. For this case, with factors from the parturient, delivery and anesthesia technique excluded, and the rapid and large precipitation of high concentration of ropivacaine with L_alk_ *in vitro*, the patient's paresthesia could potentially, but not certain, be explained by drug crystallization. Elimination *via* macrophage phagocytosis could help explain her symptom relief (9). However, this hypothesis warrants pathological examination for further confirmation.

In summary, adding L_alk_ to pretreated 0.09% ropivacaine (routine labor analgesia) was safe and efficient. We advise avoiding further high concentration of ropivacaine (0.5–1%) administration; additional dilution of lidocaine with low concentration and high volume may provide safe maintenance of anesthesia and analgesia. This finding also supports the pharmacological principle that a combination of drugs should be considered incompatible, until proven compatible.

## Data Availability Statement

The original contributions presented in the study are included in the article/supplementary material, further inquiries can be directed to the corresponding author/s.

## Ethics Statement

Written informed consent was obtained from the individual(s) for the publication of any potentially identifiable images or data included in this article.

## Author Contributions

AZ drafted the manuscript and performed the *in vitro* testing. WL and WC performed the anesthesia. YZ followed up the patient. YH helped the study design. LP conceived of the study and participated in its design. All authors have read and approved the final manuscript.

## Funding

The device, materials, and medications used in the *in vitro* testing were supported by the National Natural Science Foundation of China #81901148 (AZ) and #82071252 (YH).

## Conflict of Interest

The authors declare that the research was conducted in the absence of any commercial or financial relationships that could be construed as a potential conflict of interest.

## Publisher's Note

All claims expressed in this article are solely those of the authors and do not necessarily represent those of their affiliated organizations, or those of the publisher, the editors and the reviewers. Any product that may be evaluated in this article, or claim that may be made by its manufacturer, is not guaranteed or endorsed by the publisher.
